# Impact of Fertilizing Pattern on the Biodiversity of a Weed Community and Wheat Growth

**DOI:** 10.1371/journal.pone.0084370

**Published:** 2014-01-08

**Authors:** Leilei Tang, Chuanpeng Cheng, Kaiyuan Wan, Ruhai Li, Daozhong Wang, Yong Tao, Junfeng Pan, Juan Xie, Fang Chen

**Affiliations:** 1 Key Laboratory of Aquatic Botany and Watershed Ecology, Wuhan Botanical Garden, Chinese Academy of Sciences, Wuhan, China; 2 Hainan Modern Agriculture Inspection and Testing Precaution & Control Center, Agricultural Department of Hainan Province, Haikou, China; 3 Ecological Restoration (ECORES) Lab, Chengdu Institute of Biology, Chinese Academy of Sciences, Chengdu, China; 4 Institute of Plant Protection and Soil Science, Hubei Academy of Agricultural Sciences, Wuhan, China; 5 Institute of Soil and Fertilizer Science, Anhui Academy of Agricultural Sciences, Hefei, China; 6 China Program of International Plant Nutrition Institute (IPNI), Wuhan, China; North Carolina State University, United States of America

## Abstract

Weeding and fertilization are important farming practices. Integrated weed management should protect or improve the biodiversity of farmland weed communities for a better ecological environment with not only increased crop yield, but also reduced use of herbicides. This study hypothesized that appropriate fertilization would benefit both crop growth and the biodiversity of farmland weed communities. To study the effects of different fertilizing patterns on the biodiversity of a farmland weed community and their adaptive mechanisms, indices of species diversity and responses of weed species and wheat were investigated in a 17-year field trial with a winter wheat-soybean rotation. This long term field trial includes six fertilizing treatments with different N, P and K application rates. The results indicated that wheat and the four prevalent weed species (*Galium aparine*, *Vicia sativa*, *Veronica persica* and *Geranium carolinianum*) showed different responses to fertilizer treatment in terms of density, plant height, shoot biomass, and nutrient accumulations. Each individual weed population exhibited its own adaptive mechanisms, such as increased internode length for growth advantages and increased light interception. The PK treatment had higher density, shoot biomass, Shannon-Wiener and Pielou Indices of weed community than N plus P fertilizer treatments. The N1/2PK treatment showed the same weed species number as the PK treatment. It also showed higher Shannon-Wiener and Pielou Indices of the weed community, although it had a lower wheat yield than the NPK treatment. The negative effects of the N1/2PK treatment on wheat yield could be balanced by the simultaneous positive effects on weed communities, which are intermediate in terms of the effects on wheat and weeds.

## Introduction

Weeds are one of the major constraints to crop yields and quality [1], [2]. However, as one of the primary producers within farming systems, weeds are of central importance to the arable system's food web. The weed community provides a range of resources for higher trophic groups, supports a high diversity of insect species and birds [3], [4], and therefore plays an important role in the biological diversity of agroecosystems. Weeds also have other ecosystem functions, including nutrient cycling and soil preservation [5]. In addition, there are correlations between an impoverished landscape and the appearance of pests and diseases [6]. Moreover, an overreliance on herbicides has imposed a cost upon society and the environment [7]. The biodiversity of weed communities in a cropland can therefore be an important element for the reliable and sustainable provision of agroecosystem services. However, encouraging in-field biodiversity is unpopular among farmers because of the risk of decreased crop production as a result of weed competition. It is important to match crop production with conservation of biological resources to develop more sustainable systems [8].

Crop nutrient management practices may serve as an important component of more robust weed management programs [9]–[11]. Fertilization alters soil fertility, thus affecting weed density, nutrient uptake, and biomass yield, which in turn affects species composition and biodiversity [9], [12]–[14]. For example, Mahn [15] observed a general decline in the number of weed individuals and an increase in weed biomass with increasing rates of nitrogen (N) fertilizer. In another study, *Digitaria ischaemum* Shreb (smooth crabgrass) was found to be the dominant species under N + potassium (K) and non-fertilized treatments, *Cyperus rotundus* L (purple nutsedge) dominated under phosphorus (P)+K treatment, and more weed species and higher Shannon' s diversity (*H′*) values were detected in the balanced fertilization treatment [14]. These effects, however, indirectly depend on the light penetration caused by crop competition variation [16]. Balanced fertilization promotes the growth of crops, resulting in closed crop stands and light limitation for the weed communities growing underneath, thereby affecting weed species diversity [14], [17].

Varying physiological responses of weed species to soil amendments are one of the explanations for weed community biodiversity [18]. Murphy and Lemerle [18] reported that the type and rate of fertilizers applied and the physiology of the species involved play an important role in weed population shifts. Haas and Streibig [19] also showed that high N levels will favor weed species that possess either physiological shade tolerance (esp. *Stellaria media*) or are able to climb into more favorable light conditions (esp. *Galium aparine*). Pyšek and Lepš [20] and Bengtsson et al. [21] also found that N fertilization favors shade-tolerant, climbing, and competitive weed species, but suppresses other kinds of species and results in a decrease of weed species diversity. With cessation of mineral fertilizer application during the period of conversion in a cropland, van Elsen [6] observed a gradual decline of weeds like *G. aparine*, which need a high nitrate level, and an increase in leguminous weeds (esp. *Vicia spp*.).

Documentation of the effects of a farming system on vegetation diversity is an important step toward understanding ecosystem function in agricultural landscapes [22], but only a handful of studies have examined the effect of fertilization on weed community biodiversity in agroecosystems. Biodiversity promotes ecosystem productivity, sustainability and stability in grassland [23]–[24]. Based on these reports, it is hypothesized that appropriate fertilization of agroecosystems would not only provide desirable crop productivity but also maintain the biodiversity of weed communities.

Long-term field experiments would ensure that proper data are accumulated and that confounding experimental effects are reduced, thereby providing better insights into the effects of prolonged fertilization over time [25]. It was also hypothesized that weed species could develop physiological adaptive mechanisms under long-term fertilization conditions. This study was conducted in a winter wheat field under continuous fertilization since 1994 with the objective of examining the effects of different N, P and K fertilization patterns on crop growth and yield. Effects on weeds were not examined at initiation, so baseline data are unavailable. Therefore, the objective of this study was to evaluate the cumulative effects of different fertilizing patterns on weed community biodiversity and their adaptive mechanisms with data from one-year sampling in this long-term experiment.

## Materials and Methods

### Site and Experimental Design

The long-term fertilization field experiment used in this study has been run by the Anhui Academy of Agricultural Sciences since 1994 and is located in Mengcheng county in the Anhui province of China (33°13′38˝ N, 116°36′58˝ E). The field studies did not involve endangered or protected species and no specific permits were required. This region has a warm temperate to sub-humid monsoon climate. The mean temperature, precipitation, evaporation and selected soil properties of the experimental site are given in Table 1. Since 1999, there has been a crop rotation of winter wheat (*Triticum aestivum* L.) and soybean (*Glycine max* [L.] Merr.).

**Table 1:** Experimental conditions at the field site.

Item		Value
Weather		
Mean annual temperature (1994–2010)		15°C
Rainfall (annual mean, 1994–2009)		872 mm
Rainfall (2010)		821 mm
Evaporation (annual mean, 1994–2010)†		1026 mm
Soil conditions		
Soil type		Lime concretion black soil
Soil pH		8.0
Soil organic matter		9.9 g kg^−1^
Total soil N		0.79 g kg^−1^
Soil available P		7.8 mg kg^−1^
Soil available K		111 mg kg^−1^
Crop rotation		
Winter wheat-maize cropping system		1994–1998
Winter wheat- Soybean cropping system		1999–2010
Fertilizer treatments		N, P_2_O_5_, K_2_O kg ha^−1^
Control		0, 0, 0
PK		0, 90, 135
NP		188, 90, 0
NK		188, 0, 135
NPK		188, 90, 135
N1/2PK*		188, 45, 135

Evaporation was measured using the Penman formula.

The N1/2PK treatment was designed to further evaluate the effect of P on winter wheat as it was the most critical soil limiting nutrient at the beginning of the experiment.

Fertilizer treatments consisted of six combinations with different rates of N, P and K (Table 1) applied as urea, calcium superphosphate, and potassium chloride, respectively. All fertilizers were applied by soil surface broadcasting before the sowing of wheat. No fertilizer was applied during the soybean production seasons. The plot size was 20 m^2^ (4×5 m) with three replications in a randomized complete block design. Soybean (‘Zhonghuang 13’) and winter wheat (‘Yannong 19’) were planted at a density of 249,800 seeds and 4,685,100 plants per hectare on 15 June and 20 October 2010, respectively.

Herbicides were used according to the weed spectrum and expert recommendations. All treatments had a broad application of herbicides, and manual weeding was implemented before 2008. To better examine the effects of fertilization on weed community biodiversity, only a broadcast application of herbicides was continued in each growing season, and without manual weeding after 2008. Tribenuron methyl (Anhui Research Institute of Chemical Industry, China) at 12 g ai.ha^−1^ for wheat and Acetochlor (Anhui Huilong Group Rmf Agrochemical Co., Ltd, China) at 1500 g ai.ha^−1^ for soybean were used once every growing season in all the experimental plots.

### Sampling Procedures

Weed evaluations were conducted three times during wheat growing season on April 10 (177 days after sowing [d]), May 8 (205 d), and May 23 (221 d), 2010. At each sampling date (growing period), three 0.5×0.5 m quadrats were systematically positioned to avoid edge effects and re-sampling of the previously sampled areas in each plot. Five wheat plants and all weeds present in the quadrat were clipped, collected, sorted by species, counted, and oven dried at 70°C for 48 h before weighing. Wheat tillers were also counted, and plant heights of wheat and weed species were measured.

At 205 d and 221 d, the tallest plant of *Galium aparine* (a prevalent broadleaved weed species) in each quadrat was selected for the measurement of internode length. Light measurements were also made in the quadrats (using Minolta Illuminance Meter T-1H) above the wheat and on the soil surface. At 221 d, each individual weed species and wheat plant was sampled for total N, P and K analysis. The N was measured using the Kjeldahl method, and P and K were measured using an inductively coupled plasma optical emission spectrometer (ICP-OES) after the plant samples were digested [26].

### Statistical Procedures

Weed density was calculated as the total plant number of a particular weed species per square meter, total weed density was calculated as the total of all weed species per square meter, and winter wheat density was calculated as the total number of tillers per square meter. The species diversity of weed communities was assessed by calculating different indices. For example, Species richness (*S*) was measured by the mean number of species in each treatment [27]. Shannon's diversity index (*H*′), Simpson index (*D*) and Pielou index (*E*) were calculated for the weed communities using the following equations:
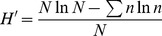


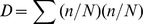


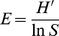
where *N* is the total number of individuals per plot and *n* is the number of individuals per species per plot. Shannon's diversity index (*H′*) takes not only the number of species (community richness) into account but also the relative distribution of each species in the community (community evenness). The Simpson index was applied to measure the degree of dominance of individual weed species.

Rank-abundance plots were used to display species relative abundance data. Abundance distributions provide a complete description of the community diversity and simultaneously show both components of species diversity, species number, and evenness. The relative abundance of a species indicates its degree of dominance or subordination in the weed community (i.e., the greater the relative abundance of a species in the weed community, the higher its dominance, [28]). Relative growth rate (RGR, g m^−2^ d^−1^), the increase of plant biomass per unit time, was used to compare crop growth with that of weed species.

Statistical analysis was done using SPSS version 16.0. Weed density and biomass data were log_e_ (x+1) transformed before analysis to meet homogeneity of variance assumptions. For normally distributed parameters, the General Linear Model Univariate was used, and means were compared based on Tukey's multiple comparison tests P<0.05. Otherwise, nonparametric tests were used, and median values were presented.

## Results

### Structural Changes and Biological Diversity at the Community Level

**Figure 1:**
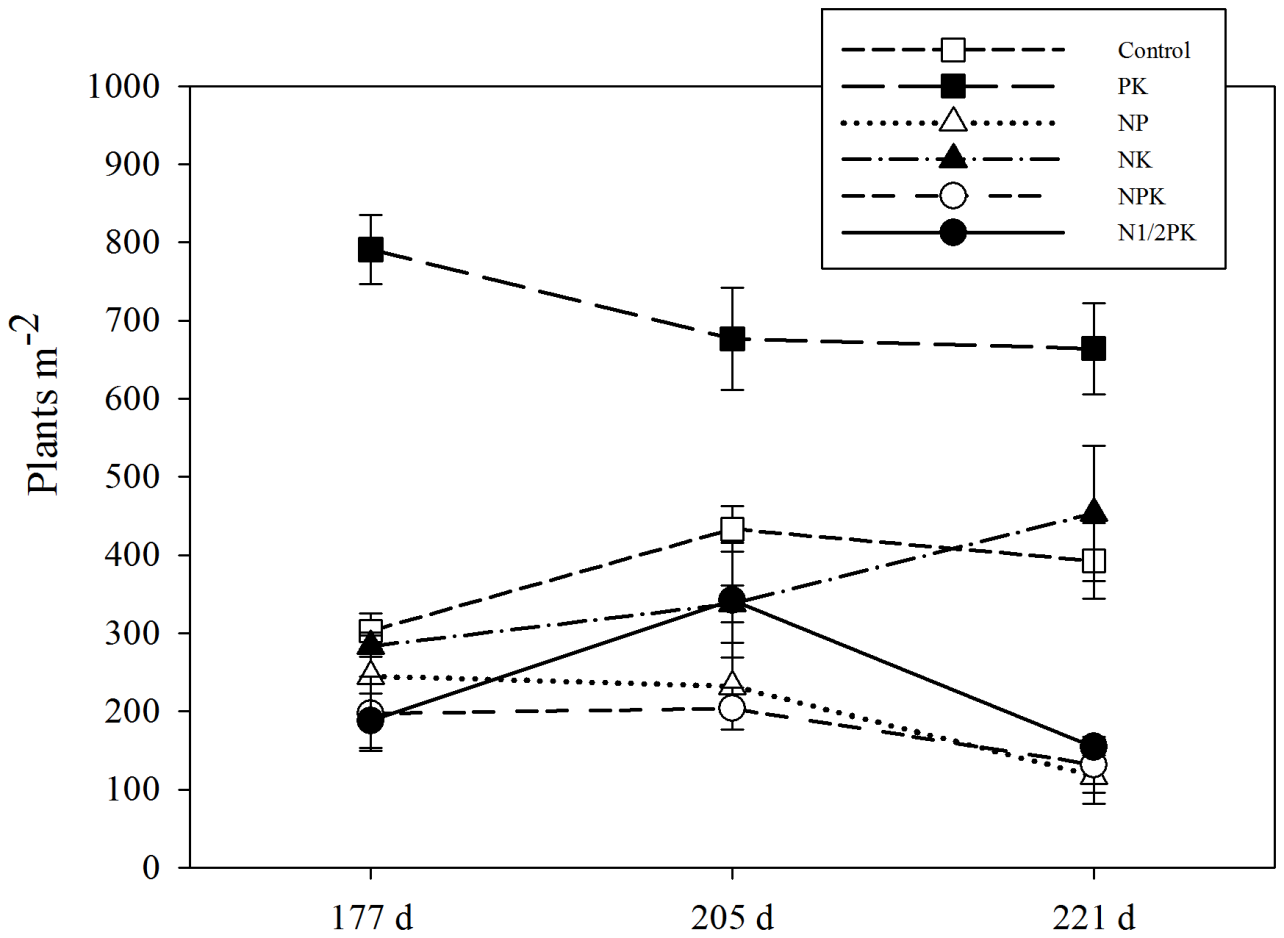
The influence of fertilization on the density of the weed community.

The results indicated that total weed density was influenced by fertilization (P<0.0001), but was not affected by growing periods (P = 0.59) (Fig. 1). A general decline in the number of weed individuals was observed in the different fertilization treatments except in the NK treatment where the number increased slightly. The PK treatment showed the highest total mean weed density (711 plants m^−2^) followed by the control and NK treatments. Weed densities in these three treatments were significantly higher than the 201 plants m^−2^ in the treatments with N and P (72% less than that in the PK treatment).

**Figure 2:**
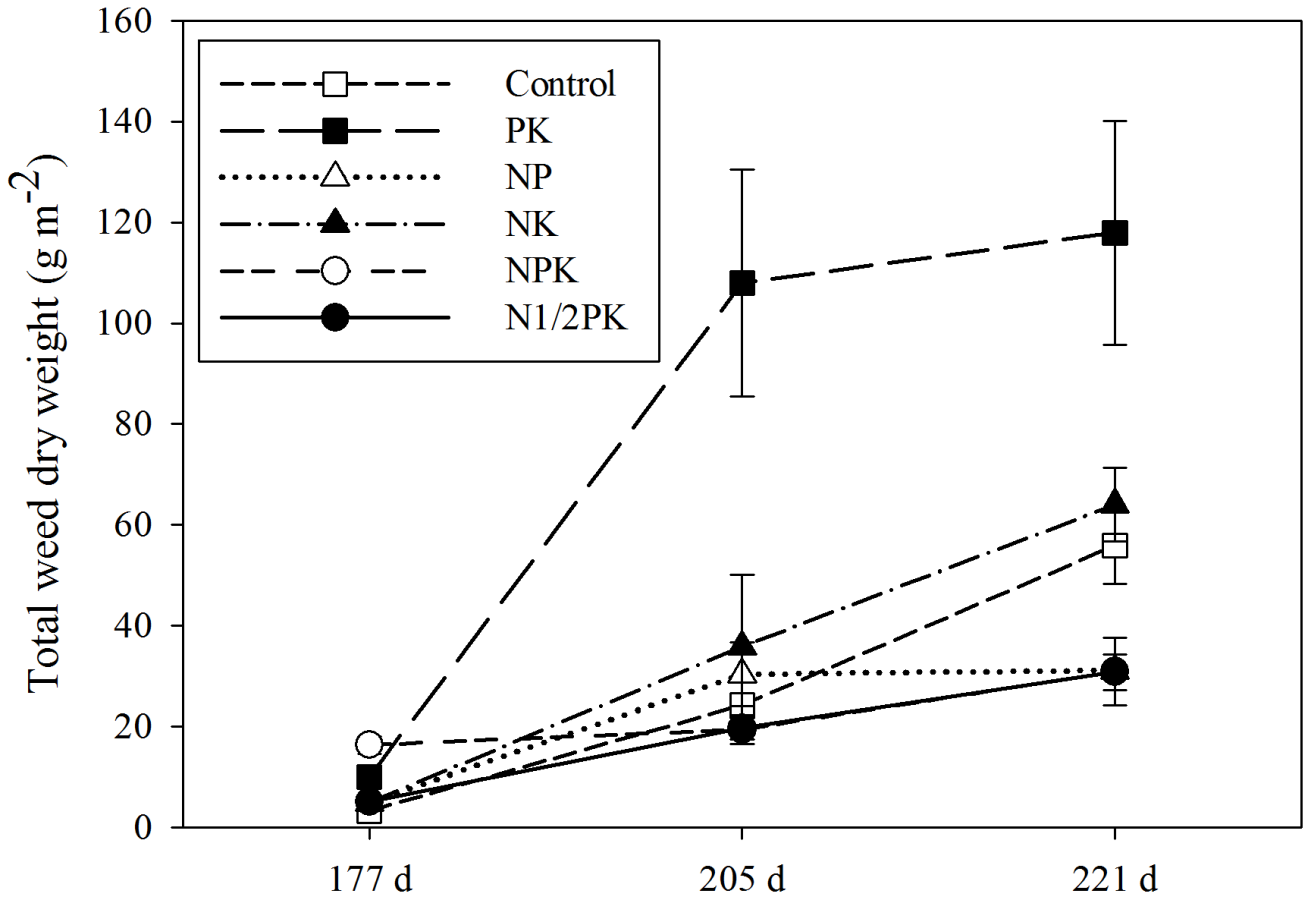
The influence of fertilization on the biomass of the weed community.

Fertilization increased weed biomass in the winter wheat field (Fig. 2). The PK treatment obtained the highest (P<0.05) total weed biomass of 108.03 and 117.94 g m^−2^ at 205 and 221 d, respectively. Treatments with N and P showed a lower weed shoot biomass.

**Figure 3:**
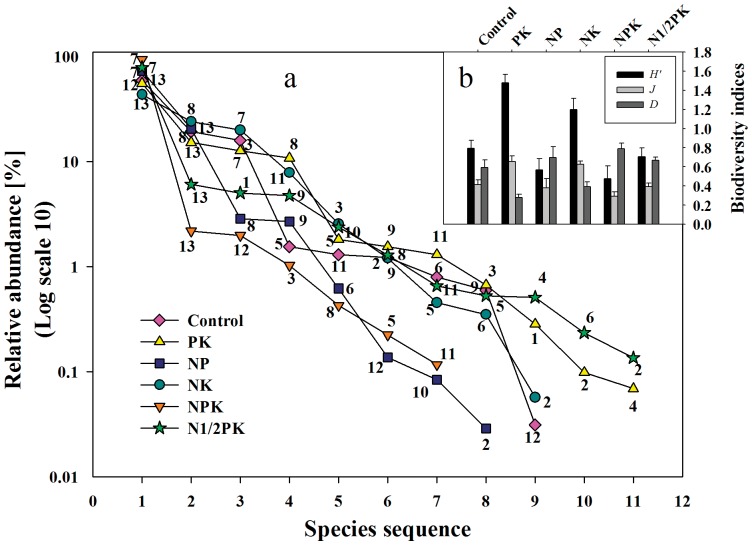
The influence of fertilization on the species diversity of the weed community. (a) Rank-abundance plots corresponding to different fertilization treatments in a winter wheat field. (b) The influence of fertilization on the biodiversity indices of weed communities (*H′*, Shannon-Winner Index; *J*, Pielou Index; and *D*, Simpson index). Species reference: (1) *Avena fatua* L., (2) *Calystegia hederacea* Wall., (3) *Cirsium segetum* Bge., (4) *Cyperus rotundus* L., (5) *Erigeron annuus* (L.) Pers., (6) *Euphorbia helioscopia* L., (7) *Galium aparine* L. var. *tenerum* (Gren.et Godr.) Rcbb., (8) *Geranium carolinianum* L., (9) *Lithospermum arvense* L., (10) *Mazus pumilus* (Burm. f) V. Steenis., (11) *Plantago virginica* L., (12) *Veronica persica* Poir., (13) *Vicia sativa* L.

The species diversity of the weed community was modified by fertilizing patterns. Species number and evenness were affected differently by the fertilization treatments, which were shown in (a) the rank-abundance plots and (b) the vertical bar chart (Fig. 3). Among all the recorded species (13 spp.), the species numbers in the PK (11 spp.) and N1/2PK (11 spp.) treatments reached the highest, followed by the control (9 spp.) and NK (9 spp.) treatments, while the lowest value (7 spp.) was observed in the NPK treatment.

The evenness of the weed community also varied with the fertilization treatments (Fig. 3a, b). Fertilizing patterns greatly shaped the equitability in the partitioning of total biomass among species in the community as shown by the slope of the rank-abundance plots. A higher Shannon-Wiener Index (Fig. 3b) and weed community evenness (Pielou Index, Fig. 3a, b) was observed in the PK treatment, followed by the NK and control treatments. However, in these treatments, the Simpson Index was lower compared with that in the NP and NPK treatments (Fig. 3b). Compared with the NPK treatment, the half P rate treatment (N1/2PK) showed a higher equitability of total biomass partitioning (Fig. 3a) with higher Pielou Index and Shannon-Wiener Index values (Fig. 3b).

### Changes at the Population Level

#### Densities of Weed Population

**Figure 4:**
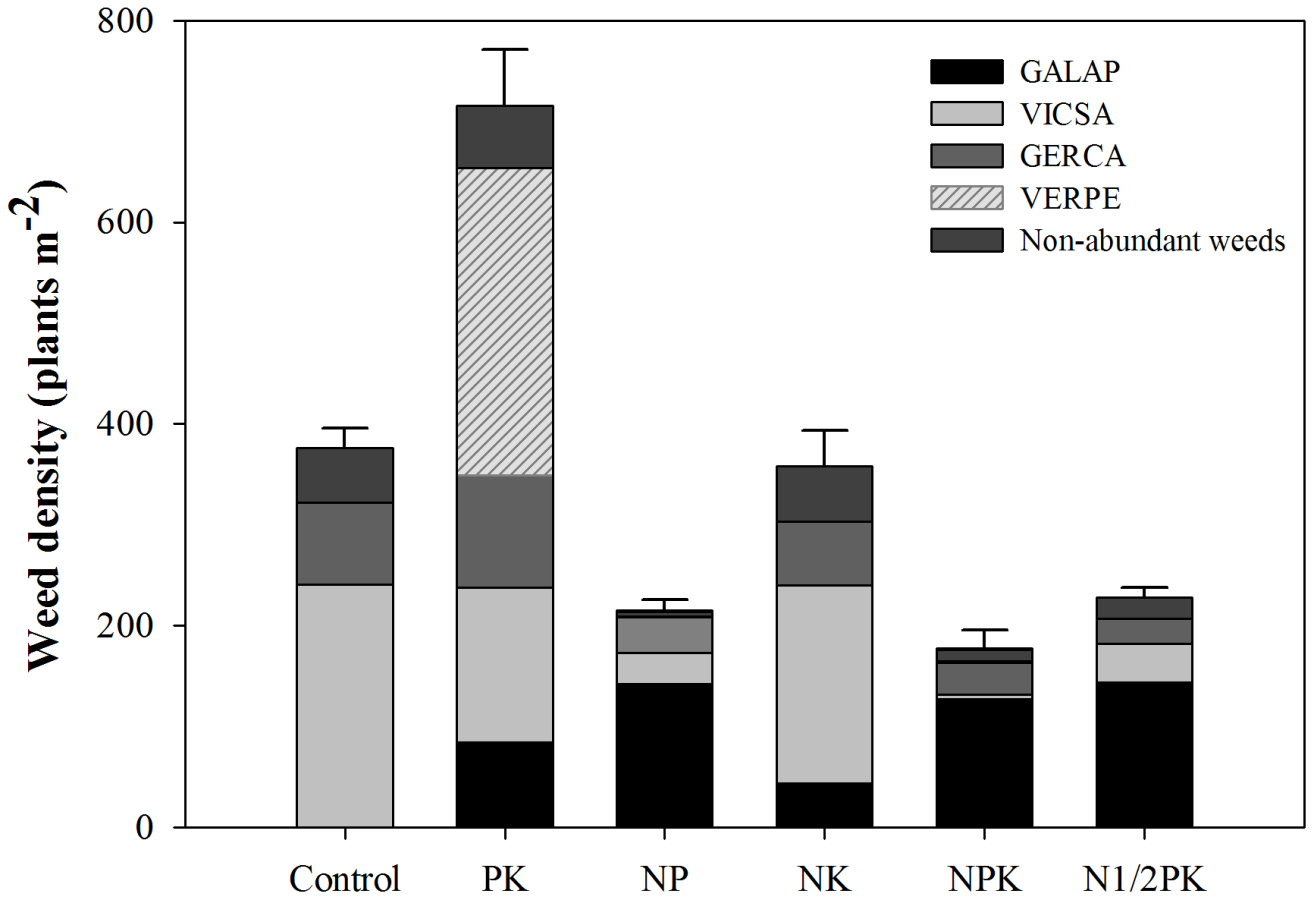
The effects of fertilization on average weed densities. Analyses of variance were performed on log_e_-transformed data, but means of untransformed data are shown. Error bars are the SE of the mean.

*Galium aparine* L. (tender catchweed bedstraw), *Vicia sativa* L. (common vetch), *Veronica persica* Poir (iran speedwell), and *Geranium carolinianum* L. (carolina geranium) were the most dominant species in the weed community. These four dominant species comprised >90% of the total weed density. Average densities of the four prevalent broadleaved weeds during the three growing periods in the different fertilization treatments are shown in Fig. 4. Densities of these four weed species varied significantly among the treatments. *Galium aparine* densities were greatly affected by fertilization (P<0.0001) and growing period (P = 0.02). No significant difference was detected in the interactions of treatments with growing period (P = 0.43). The densities of *G. aparine* (138 plants m^−2^) were higher in the treatments with N and P fertilizers, while no *G. aparine* was found in the control treatment.

*Vicia sativa* density was significantly affected by fertilization (P<0.0001) and growing period (P = 0.008). However, in contrast to *G. aparine*, *Vicia sativa* grew better in treatments without N and/or P with an average density of ∼200 plants m^−2^, while it had only 10–30 plants m^−2^ in the treatments with N and P. *Geranium carolinianum* density was also affected by fertilization (P<0.0001) and growing period (P<0.001), and the interactions of fertilization with growing period (P<0.001). Only three plants of *G. carolinianum* were found in the NP treatment at 205 d and no plants were found at 221 d. During the early growing period, *G. carolinianum* densities showed no significant differences among the treatments, with an average value of 72 plants m^−2^. However, its densities responded differently to fertilization treatments in the latter growing period, with the same tendency as *V. sativa* in each treatment. *Veronica persica* grew well only in the PK treatment, and few or no plants were detected in the other treatments.

#### Plant Height of Weed Population

**Figure 5:**
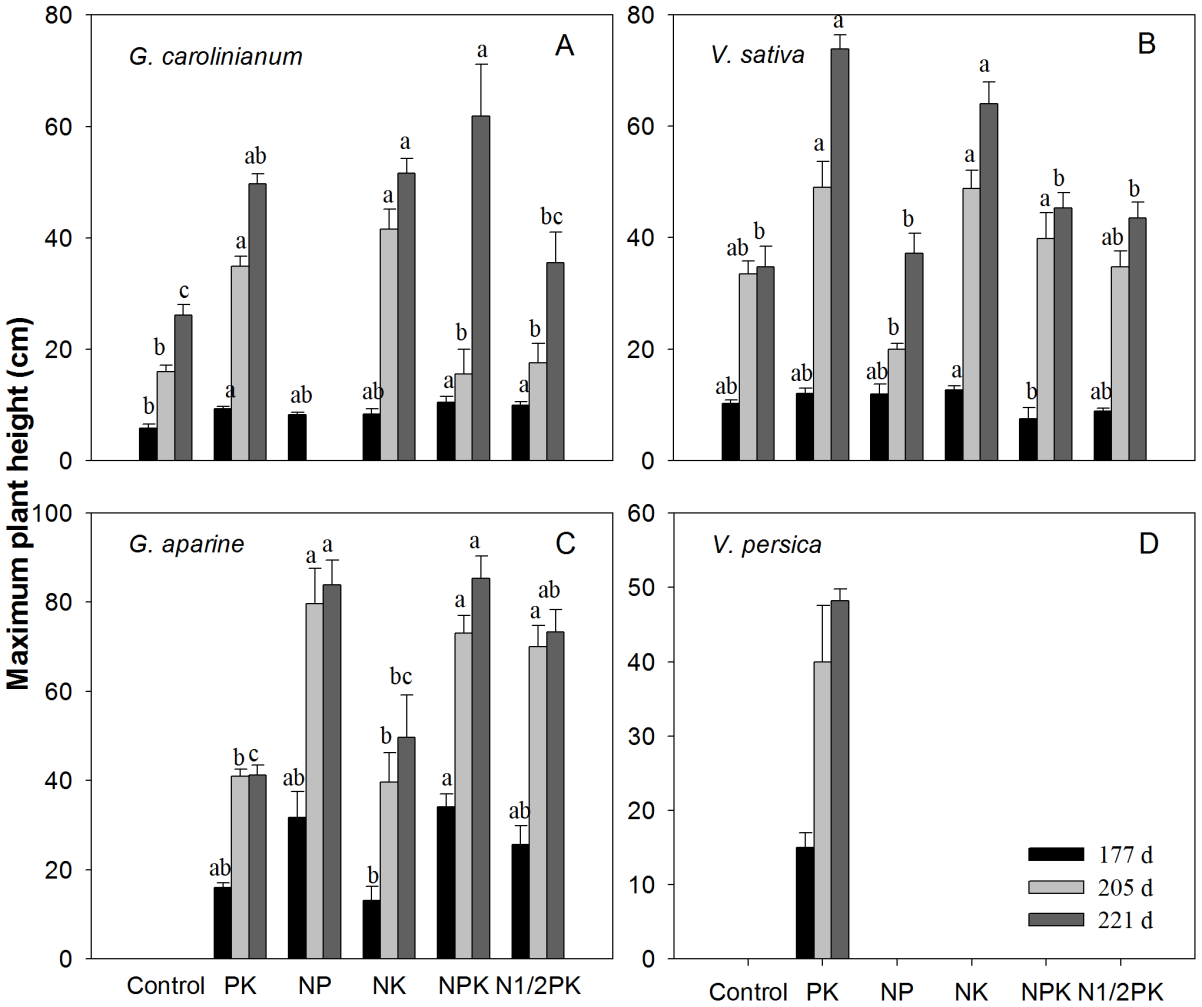
The influence of fertilization on plant height of *G. carolinianum* (A), *V. sativa* (B), *G. aparine* (C) and *V. persica* (D).

Among the four prevalent broadleaved weed species, *V. persica* grew underneath the crop canopy, and its maximum plant height was <50 cm (Fig. 5D). On the contrary, other species were erect for capturing adequate light. Fertilization significantly affected plant height of *G. carolinianum* (Fig. 5A), *V. sativa* (Fig. 5B) and *G. aparine* (Fig. 5C). *Geranium carolinianum* was taller in the PK and NK treatments, even taller than in the NPK treatment at 205 d. Plant height of *V. sativa* in the PK treatment was at a maximum, reaching 73.8 cm at maturity stage, followed by the NK treatment where it reached 64.1 cm, while lower values were observed in treatments with N and P. Conversely, *G. aparine* showed higher plant heights of 83.8 cm, 85.3 cm and 73.3 cm in the NP, NPK and N1/2PK treatments, respectively. The study confirmed that fertilization had little effect on the number of *G. aparine* shoot internodes. However, fertilization significantly modified the internodal length of *G. aparine*, especially from the 4^th^ to the 12^th^ internode (Table 2).

**Table 2:** Effects of different fertilization treatments on internode length (cm) and number of *G. aparine* plant.

Treatment	Internode number	The 4^th^ Internode	The 5^th^ Internode	The 6^th^ Internode	The 7^th^ Internode	The 8^th^ Internode	The 9^th^ Internode	The 10^th^ Internode	The 11^th^ Internode	The 12^th^ Internode
177 d										
CK	–	–	–	–	–	–	–	–	–	–
PK	9 a	1.5 b	2.0 ab	2.1 bc	2.1 b	2.0 bc	1.3 b	–	–	–
NP	11 a	1.8 b	2.5 ab	2.8 ab	4.3 a	3.9 ab	3.2 ab	–	–	–
NK	9 a	1.4 b	1.6 b	1.4 c	1.6 b	1.5 c	1.0 b	–	–	–
NPK	11a	2.8 a	3.1 a	2.8 ab	2.5 ab	4.0 ab	5.5 a	–	–	–
N1/2PK	10 a	2.4 a	2.9 a	3.2 a	3.3 ab	3.3 a	3.4 ab	–	–	–
205 d										
CK	–	–	–	–	–	–	–			
PK	16 a	1.4 b	1.9 b	2.5 c	3.2 c	3.8 b	4.7 bc	4.7 ab	4.3 bc	4.4 b
NP	18 a	3.8 ab	4.4 ab	5.8 ab	6.1 ab	6.3 a	6.2 abc	5.9 ab	6.1 ab	5.1 ab
NK	16 a	2.6 ab	3.0 ab	3.6 bc	4.1 bc	4.2 b	4.2 c	3.8 b	3.7 c	3.4 b
NPK	18 a	3.7 a	4.9 a	6.2 a	7.0 a	7.2 a	6.9 a	6.1 a	6.7 a	6.8 a
N1/2PK	18 a	3.3 ab	4.3 ab	5.2 ab	6.1 ab	6.4 a	6.3 ab	6.0 a	5.8 ab	5.3 ab

Different letters within each day after sowing (d) indicate significant differences among treatments (Tukey test; P<0.05).

The internode lengths (heights) are taken between the previous node and the listed node.

#### Shoot Biomass of the Weed Population

Shoot biomass of the four weed species varied with different fertilization treatments (Table 3). Like the weed density, *G. aparine* shoot biomass in the NP treatment was highest at each growing period, followed by the NPK and N1/2PK treatments. The relative growth rates of *G. aparine* in the treatments without N and/or P were lower, especially in the NK treatment (0.11 g m^−2^ d^−1^), than that in the NP treatment (0.83 g m^−2^ d^−1^). The highest proportion of *G. aparine* shoot biomass to total weed biomass was obtained in the NPK treatment (91%), followed by the NP and N1/2PK treatments at 89% and 77%, respectively, while treatments without N and/or P had the lowest value (8%). These results indicated that *G. aparine* had an absolute growth advantage under higher soil fertility conditions, and its growth could be significantly promoted by the application of N and P fertilizers.

**Table 3:** Shoot biomass of the four prevalent weed species at 177, 205 and 221

Treatments	Weed biomass (g m^−2^)			Biomass proportion (%)^e^		Nutrient uptake (g m^−2^)		
	177 d	205 d	221 d	205 d	221 d	N	P	K
*G. aparine*								
Control	0.00	0.00	0.00	0.00	0.00	—	—	—
PK	1.39 ab	10.38 bc	10.38 bc	12.72 b	8.80 b	0.066 b	0.019 c	0.085 b
NP	2.85 a	27.05 a	27.63 a	89.06 a	89.13 a	0.331 a	0.093 ab	0.337 b
NK	0.85 b	2.68 c	5.21 c	7.48 b	8.14 b	0.243 ab	0.035 c	0.276 b
NPK	2.83 a	18.24 ab	27.33 a	94.03 a	88.41 a	0.455 a	0.114 a	0.611 a
N1/2PK	2.43 a	15.43 ab	23.42 ab	78.41 a	76.02 a	0.273 ab	0.064 ab	0.392 ab
*V. sativa*								
Control	2.68 ab	16.42 a	22.75 a	67.71 a	40.72 a	0.365 a	0.042 b	0.352 a
PK	3.73 a	12.41 a	28.58 a	15.20 b	24.24 a	0.538 a	0.133 a	0.400 a
NP	0.48 bc	0.47 b	1.53 b	1.56 c	5.26 b	0.028 b	0.006 c	0.013 b
NK	3.54 a	15.60 a	24.17 a	43.55 a	37.76 a	0.442 a	0.061 b	0.412 a
NPK	0.11 c	0.42 b	0.64 b	2.19 c	2.07 b	0.009 b	0.002 c	0.006 b
N1/2PK	1.31 bc	1.19 b	1.83 b	6.06 bc	5.94 b	0.035 b	0.006 c	0.032 b
*G. carolinianum*								
Control	0.65 b	4.68 abc	8.68 abc	19.30 a	15.53 ab	0.124	0.016	0.093
PK	5.59 ab	8.83 a	16.42 a	10.82 ab	13.93 abc	0.172	0.107	0.253
NP	7.50 ab	1.06 bc	0.00 d	3.50 ab	0.00 c	—	—	—
NK	2.94 ab	8.62 ab	12.94 ab	24.07 a	20.22 a	0.200	0.034	0.214
NPK	9.11 a	0.08 c	1.75 bcd	0.43 b	5.65 bc	0.024	0.008	0.027
N1/2PK	4.53 ab	0.25 d	0.28 cd	1.29 ab	0.26 c	0.001	0.000	0.002
*V. persica*								
Control	0.00	0.00	0.00	0.00	0.00	—	—	—
PK	4.68	65.94	81.19	59.69	58.52	0.762	0.415	1.903
NP	0.00	0.00	0.68	0.00	2.25	—	—	—
NK	0.00	0.00	0.33	0.00	0.51	—	—	—
NPK	0.01	0.13	0.12	0.81	0.30	—	—	—
N1/2PK	0.00	0.00	0.00	0.00	0.00	—	—	—

^e^ indicates the proportion of shoot biomass within a species to the total weeds biomass per treatment.

Different letters within a species indicate significant differences among treatments (Tukey test; P<0.05).

Biomass accumulation of *V. sativa* followed an opposite trend to that of *G. aparine* for the different fertilization treatments. The average shoot biomass of *V. sativa* in the control, NK and PK treatments were 3.32, 14.81, and 25.17 g m^−2^, respectively in contrast to values under the NP, NPK and N1/2PK treatments where the values were 0.63, 0.69, and 1.33 g m^−2^ at 177, 205 and 221 d, respectively. The relative growth rate showed that *V. sativa* also grew rapidly in the control, PK and NK treatments (0.50 g m^−2^ d^−1^). The highest proportion of *V. sativa* shoot biomass to the total weed biomass was observed in the control treatment with 68% and 41% at 205 and 221 d, respectively, followed by the NK and PK treatments, while treatments incorporating N and/or P fertilizers showed the lowest values (4%).

The average shoot biomass of *G. carolinianum* at 177 d reached 5.05 g m^−2^, which was much higher than the corresponding values for *G. aparine*, *V. sativa* and *V. persica* (1.72, 1.97 and 0.55 g m^−2^, respectively). The *G. carolinianum* shoot biomass under the NPK treatment (9.11 g m^−2^) was higher than that under control. However, in the middle and latter growing periods, *G. carolinianum* shoot biomass performed similarly to *V. sativa* with the different fertilization treatments. Specifically, biomass accumulation of *V. persica* was only higher in the PK treatment. Its biomass at 205 d accounted for 81% of the total biomass at 221 d, and it also accounted for >50% of the total shoot biomass of weeds present in the PK treatment.

#### Nutrient Uptake by Weeds

Nutrient uptake by the four weed species differed among treatments (Table 3). In general, nutrient uptake was higher in *G. aparine* and *V. sativa* than in *G. carolinianum*, suggesting that the two weed species were stronger nutrient competitors to wheat. Nutrient uptake of weed species in different treatments followed a pattern similar to shoot biomass in the corresponding treatments. Average N, P, and K uptakes by *G. aparine* in the NP, NPK, and N1/2PK treatments were 0.353, 0.090, and 0.447 g m^−2^, respectively, which were 435, 375, and 425% higher than those under the PK treatment, and 45, 158, and 62% higher than those under the NK treatment. The N, P, and K uptakes under the control, PK, and NK treatments were 0.426, 0.076, and 0.361 g m^−2^, respectively for *V. sativa* and 0.163, 0.052, and 0.187 g m^−2^, respectively for *G. carolinianum*. For *V. persica*, the highest nutrient uptake was found under the PK treatment, and its P and K uptakes were 127 and 241% higher, respectively than those of *V. sativa*.

### Winter Wheat Responses to Fertilization

#### Winter Wheat Density

Significant differences in wheat density were found among the fertilization treatments (P<0.001). Wheat density increased in the treatments that included N and P. Wheat density in the NP treatment was significantly (P<0.01) higher than that in the control, PK and NK treatments. However, there were no significant differences in winter wheat density within the NP, NPK and N1/2PK treatments. The average density was 524 tillers per m^2^ in the latter three treatments, which was 82% higher than that in the control treatment (data not shown).

#### Plant Height of Winter Wheat

**Figure 6:**
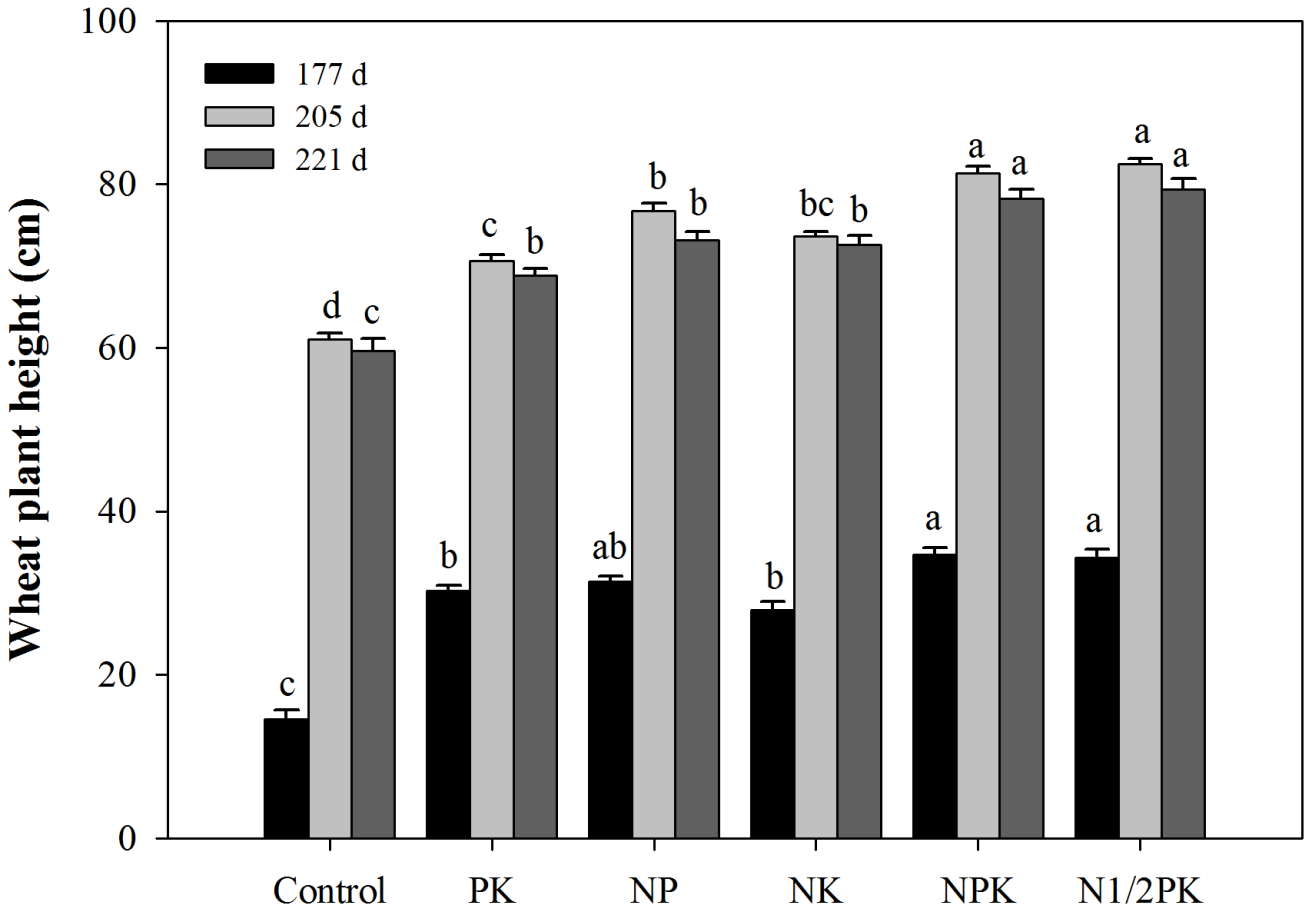
The influence of fertilization on wheat plant height at three growing periods.

Fertilization significantly increased wheat height at all the growing periods and the final heights ranged from 60.0 to 79.4 cm at maturity stage (Fig. 6). Wheat heights of the NPK (78.2 cm) and N1/2PK treatments (79.4 cm) were 31 and 33% higher, respectively than the control treatment.

#### Winter Wheat Biomass, Nutrient Uptakes and Grain Yield

Fertilization significantly increased the shoot biomass accumulation of winter wheat (Table 4). Higher wheat shoot biomasses were observed in the treatments with N and P. The highest wheat shoot biomass was found in the N1/2PK treatment, and significant differences were also found in two of the three growing periods within the treatments containing N and P.

**Table 4:** The effects of different fertilization treatments on wheat shoot biomass at 177, 205 and 221

Treatments	Biomass (g m^−2^)			Nutrient uptake (g m^−2^)			Weed parameters percentages (%)^f^				
	177 d	205 d	221 d	N	P	K	weed biomass	N	P	K	Weed number
Control	149 d	405 c	457 d	4.11 c	0.62 d	3.05 d	47.37	52.59	11.59	22.47	81.8
PK	411 c	1003 b	1032 c	5.12 c	2.37 bc	5.56 cd	100.00	100.00	100.00	100.00	100.0
NP	512 bc	1302 a	1503 b	14.52 a	4.10 a	8.12 bc	26.39	25.19	14.49	12.73	72.7
NK	395 c	843 b	1069 c	6.91 bc	1.08 cd	6.02 cd	54.27	65.93	18.84	32.96	81.8
NPK	601 ab	1337 a	1556 b	12.68 ab	3.75 ab	11.05 ab	26.21	36.30	18.84	23.97	63.6
N1/2PK	700 a	1476 a	2234 a	17.17 a	3.26 ab	14.46 a	26.12	25.19	11.59	17.60	100.0

^f^ indicates the percentage of the value of a treatment to that in the PK treatment.

Different letters indicate significant differences among treatments (Tukey test; P<0.05).

Wheat nutrient uptake is usually positively correlated with its shoot biomass accumulation. However, the wheat P uptake had no significant difference between the N and P treatments in this study (Table 4). The highest N and K uptakes were in the N1/2PK treatment. They were 18 and 78% higher than those in the NP treatment and 35 and 31%, higher respectively than those in the NPK treatment. These results might be attributed to the lower shoot biomass and nutrient accumulations of the weed community in the N1/2PK treatment (Fig. 2, Table 4). It also suggested that reducing P application in the NPK treatment decreased the productivity of weeds, such as *G. aparine* (Table 3), and improved nutrient uptake and biomass accumulation of wheat.

**Figure 7:**
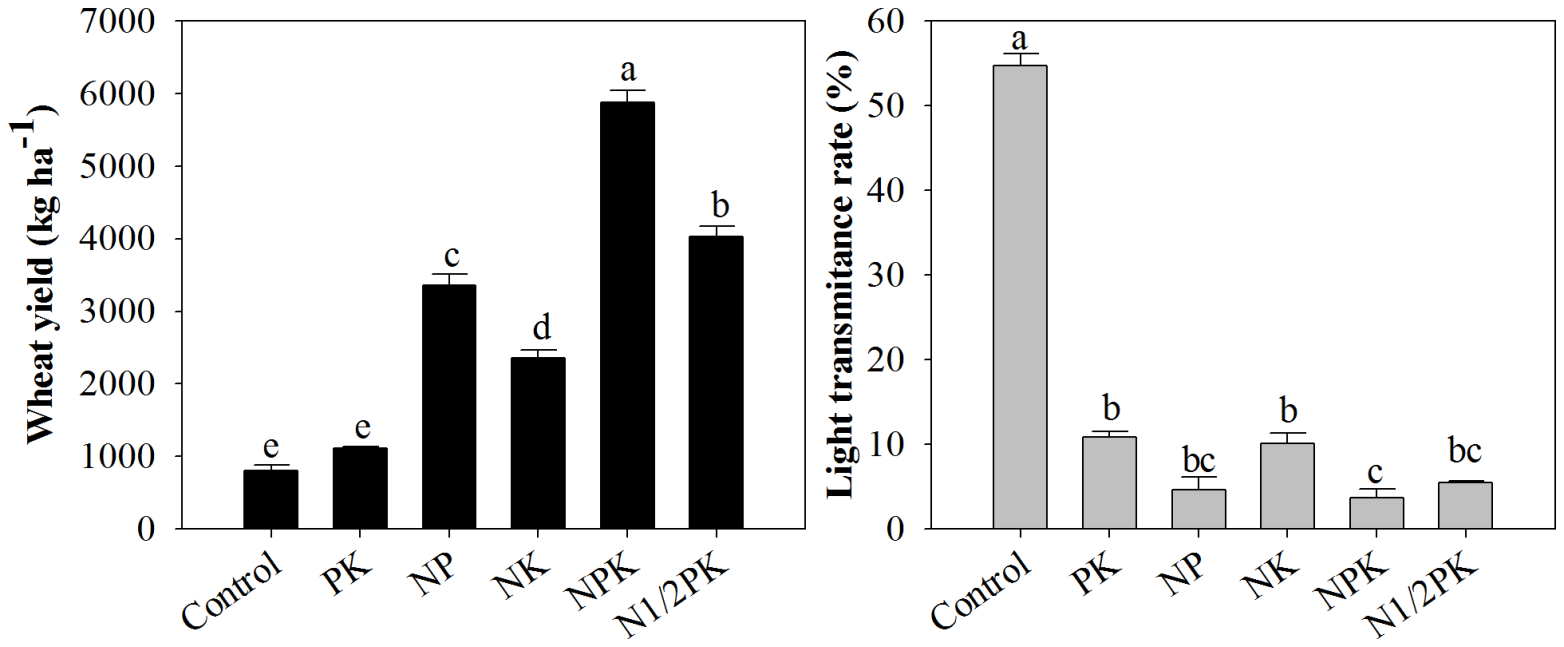
The influence of fertilization on wheat yield and light transmittance at the ground surface.

The NPK treatment resulted in the highest wheat yield, followed by the N1/2PK treatment (Fig. 7). In contrast, the control treatment obtained the highest light penetration (55%). The average light transmittance rate in the treatments with N and P was 5% at 205 d, which was 92% lower than the light transmittance value of the control treatment (Fig. 7).

## Discussion

Weed community structure changed sharply among the different fertilization treatments and the results concurred with previous research conducted under field conditions by Mahn [15]. The PK treatment showed the highest weed density and shoot biomass, followed by the NK and control treatments, while the treatments with N and P resulted in lower values. Yin et al. [14] and Nie et al. [17] also reported similar results. The structural variability of the weed community across treatments could be directly explained by the different soil fertility conditions. For example, soil available N concentration plays an important role in terminating dormancy by removing germination constraints and promoting the emergence of seedlings [29]. Soil available P was the primary nutrient regulating the species composition and floristic construction of the weed communities, followed by N and K [30]. Moreover, solar radiation reaching weeds was indirectly modulated by different fertilization treatments (Fig. 7). Wheat growth was promoted in the treatments with N and P, especially in the balanced fertilization treatments (Table 4, Fig. 7), increasing its ability to intercept solar radiation (Fig. 7), and thereby affecting the growth of weed species. The structural variability of the weed community may also be significantly affected by rainfall and other environmental factors, such as mean annual temperature. Ochoa-Hueso and Manrique [31] showed that apart from nutrients, plant germination and growth are also influenced by water availability. Pal et al. [32] and Šilc et al. [33] also reported that environmental factors, such as mean annual precipitation and temperature showed significantly positive and negative correlation coefficients with weed species composition, respectively.

The four prevalent weed populations and wheat differed substantially in their responses to different fertilization treatments. *Galium aparine* grew well in high fertility soil conditions, and was taller than wheat. *Vicia sativa* and *G. carolinianum* grew well under the control, NK, and PK treatments, and they were also taller than wheat in these treatments. *Veronica persica* grew well only in the PK treatment. These results show that the different weed species had formed their own adaptive mechanisms. Morphological plasticity enables plants to cope with a wide variety of ecological conditions, and nutrient availability has been widely shown to induce important anatomical responses [34]. For instance, *G. aparine* grew well under higher soil fertility conditions through climbing into more favorable light conditions by internode elongation (Table 2), and responded to soil nutrient supply in the order of P>N>K. In contrast, *V. sativa* and *G. carolinianum* increased their heights for receiving more light under lower soil fertility and limited nutrient conditions. Li et al. [35] reported similar results for *G. carolinianum*, but Yin et al. [13] reported different results for *G. aparine* versus *V. sativa* and *V. persica*. These differences in the effects of fertilization on cropland weeds might be due to herbicide usage. A broadcast application of herbicides was used by Li et al. [35], while no herbicide was used by Yin et al. [13]. Furthermore, environmental conditions, such as plot location, temperature, crop type, precipitation, soil texture, neighboring habitat, and soil pH, which vary in time and space, may also cause variation in weed species responses [36]–[38].

The PK treatment showed the highest Shannon-Wiener and Pielou Indices, followed by the control and NK treatments. In contrast, these three treatments had lower Simpson Indices than the treatments with N and P. Solar penetration on the ground surface was also one of the reasons for the variation in weed community biodiversity, which is similar to the findings of other studies [16], [39], [28]. Biomass is often used as an indicator of the amount of resources captured by a crop. Higher biomass and grain yield of wheat in the NP, NPK and N1/2PK treatments indicated that wheat intercepted a larger proportion of light radiation (Fig. 7), thereby reducing the amount reaching the weeds. Wheat yield showed a significant negative correlation with light penetration (Pearson correlations >0.880, P<0.0001). The species richness, Shannon-Wiener and Pielou Indices and the Simpson Index of weed communities were proved to have significantly negative and positive linear function relationships with wheat yield [30]. Shading leads to thinning mortality in individuals of subordinate weed species, and consequently reduces evenness and biodiversity among weed species [39], [40]. Only those weed species which possess either physiological shade tolerance or are able to climb into more favorable light conditions could grow well [18]. In contrast to other species, *G. aparine* had higher plant density, shoot biomass and nutrient accumulations in the NP, NPK and N1/2PK treatments with lower light penetration. Mahn [15] also reported that the mean biomass values for *G. aparine* in higher N fertilization treatments within a spring barley field were almost two times more than the values under the no N treatment. Under high soil fertility conditions in this study, *G. aparine* could enhance its plant height through internode elongation, especially from the 4^th^ to the 12^th^ internodes for capturing more sun light. Its plants were 14% and 9% taller than wheat plants in the NP and NPK treatments, respectively (Fig. 5). Therefore, weed vegetation under higher soil fertility conditions was predominantly composed of a few species, such as *G. aparine*, resulting in a higher Simpson Index and lower Pielou and Shannon-Wiener Indices.

More weed biomass and nutrient accumulations were detected in the PK treatment. Weed biomass under the NP, NPK and N1/2PK treatments was 26.4, 26.2, and 26.1%, respectively, of that under the PK treatment. Similarly, the N, P and K accumulations, as a proportion of that in the PK treatment, were 25, 14, and 13%, respectively in the NP treatment, 36, 19, and 24%, respectively in the NPK treatment and 25, 12, and 18%, respectively in the N1/2PK treatment (Table 4). These results indicated that fewer resources were captured by the weed community in the treatments with N and P, especially in the N1/2PK treatment. If a single weed species has a shoot biomass to total weed biomass ratio >10%, it is regarded as a dominant weed species. More than two dominant weed species were found under the control, PK and NK treatments, with four species present in the PK treatment. Only one dominant weed species (*G. aparine*) was present in the balanced fertilization treatment. Thus, the results also indicated that balanced fertilization could not only maintain a stable crop yield, but also greatly reduce weed productivity.

Lower solar radiation under high soil fertility conditions resulted in fewer weed species compared with the control and PK treatments. However, the number of weed species was only 73% and 64% in the NP and NPK treatments, respectively and their number in the N1/2PK treatment was the same as in the PK treatment (Table 4). These results demonstrated that *G. aparine* is sensitive to soil P, and reducing the P application rate indirectly improved the growth of other weed species. Blackshaw and Brandt [41] also reported that the competitiveness of the high P-responsive species was progressively improved as the P dose increased. Likewise, with a lower P dose in the N1/2PK treatment, its competitive ability would decrease and more opportunities would be available for other species. The N1/2PK treatment could be considered as a balanced fertilization treatment in adjusting the interactions of wheat and weeds in agroecosystems. Although wheat yield in the N1/2PK treatment was 31% less than that in the NPK treatment, its weed species number, Shannon-Wiener and Pielou Indices were significantly higher than those in the NPK treatment. It is noteworthy that a certain amount of *G. aparine* would have a high biodiversity value for invertebrates and is important for seed-eating birds [22]. Thus, applying half the P rate in the NPK treatment could be considered as a better strategy to provide desirable farming benefits and maintain the biodiversity of weed communities. The negative effects on wheat yield could be balanced by the simultaneous positive effects of reduced herbicide usage. Appropriate combinations and rates of fertilization would not only be helpful for keeping desirable crop productivity, but also maintaining the biodiversity of weed communities in an agroecosystem.
